# The Role of a Physician-Staffed Helicopter in Emergency Care of Patients on Isolated Danish Islands

**DOI:** 10.3390/healthcare9111446

**Published:** 2021-10-26

**Authors:** Alice Herrlin Jensen, Asger Sonne, Lars S. Rasmussen

**Affiliations:** 1 Centre of Head and Orthopaedics, Department of Anaesthesia, Rigshospitalet, University Hospital of Copenhagen, 2100 Copenhagen, Denmark; asger.sonne@regionh.dk (A.S.); lars.simon.rasmussen.01@regionh.dk (L.S.R.); 2 Department of Clinical Medicine, University of Copenhagen, 2200 Copenhagen, Denmark

**Keywords:** physician-staffed helicopter, HEMS, air ambulance, emergency care

## Abstract

Emergency calls may lead to the dispatch of either ground ambulances or helicopter emergency medical services (HEMS). For residents on isolated islands, the HEMS can reduce the time to hospital admission and lead to improved outcomes. This study investigated the emergency care for residents on isolated islands with a focus on the role of a physician-staffed helicopter. The data were obtained from Danish national registries and databases. We included data on emergency calls from isolated islands from the time of emergency call to discharge. We identified 1130 emergency calls from which 775 patients were registered with a hospital admission. Of these, 41% were transported by the HEMS and 36% by a ground ambulance. The median time to admission was 83 min (IQR 66–104) and 90 min (IQR 45–144) for the HEMS and ground ambulance, respectively (*p* = 0.26). The overall 30-day mortality was 6.2% (95% CI: 4.6–8.1%), and 37% of all the patients were admitted to the hospital with an unspecified diagnosis. The emergency calls from isolated islands led to the dispatch of the HEMS in 41% of the cases. The use of the HEMS did not significantly reduce the time to admission but was used in a greater proportion of patients with an acute cardiac disease (66%) or stroke (67%).

## 1. Background

The expert prehospital care and fast admission to the hospital can be important for patients with acute illness or injury. The emergency medical dispatch centers (EMDC) dispatch appropriate resources from the emergency medical service (EMS), such as a ground ambulance or a helicopter emergency medical service (HEMS), that may provide rapid retrieval with expert prehospital care depending on the staffing [1,2,3,4]. 

For patients in rural areas, including islands, which cannot be reached by road, the prolonged time of transport to the hospital may justify the more frequent use of emergency aeromedical care when time is an important factor [5,6,7,8,9,10]. 

The primary aim of this study was to describe the emergency care for residents on the isolated Danish islands. Secondly, we aimed to assess the role of a physician-staffed helicopter in time-critical conditions, time from dispatch to hospital admission, and outcome for these patients.

## 2. Material and Methods

### 2.1. Study Design

This was a retrospective observational study based on data from 1 October 2014 until 31 March 2016.

We analysed data obtained from the five Danish regions’ (Capital Region of Denmark, Region Zealand, Region of Southern Denmark, Central Denmark Region, Region of Northern Denmark) EMDCs. From the electronic logs of the EMDC, we identified emergency calls from a predefined list of 48 Danish islands based on address and postal code.

Data regarding HEMS missions were retrieved from the database HEMS file [11]. Finally, we obtained data from the Danish National Patient Registry (NPR) and the Civil Person Registry on the patients we identified from the EMDCs. Data were merged using the patients’ Civil Person Registration (CPR) number, as well as the date of emergency call (+1 day to account for inaccuracies in registration). The CPR-number is a unique identifier assigned to all Danish residents [12]. It was not possible to retrieve data on patients with invalid or missing CPR numbers. Diagnoses were categorized in groups based on definitions according to the ICD-10 (International Classification of Diseases) diagnosis codes. Unspecified diagnoses were conditions that did not fit into any overall groups. 

### 2.2. Operational Setting

The EMDCs are responsible for dispatching the appropriate resource with the right level of urgency. The appropriate resource for transportation from the scene to hospital may either be a ground ambulance or the HEMS. Ground ambulances are staffed with emergency medical technicians or paramedics and can be supplemented by a consultant anaesthesiologist in severe cases. The HEMS are staffed with a consultant anaesthesiologist, a paramedic, and a pilot, and are functioning 24 h every day of the year. The national HEMS organization was introduced on the 1 October 2014 and had three identical helicopters at the time of our study, each with its own base in Denmark. This enables the physician-staffed helicopter to reach the geographically remote parts of the country within 30 min. The HEMS are dispatched based on a range of nation-wide criteria for immediate dispatch, which are as follows: if the patient is suspected to suffer from a time-critical condition or a severe injury; by request of an ambulance crew; inter-hospital transfers; and patients located on the isolated Danish islands.

### 2.3. Statistics

Data obtained from the five EMDCs and HEMSfile were collected for each region and integrated in an Excel spreadsheet. Further descriptive statistics were performed in a licensed RStudio Version 1.3.1073 (RStudio Team (2020). RStudio: Integrated Development for R. RStudio, PBC, Boston, MA, USA).

Continuous variables were reported as medians with the interquartile range between the 1st and 3rd quartile, and groups were compared using Mann-Whitney U’s test. Categorical variables were reported using numbers and percentages with 95% confidence interval (CI) and compared with Fisher’s exact test. *p* < 0.05 was considered statistically significant. 

## 3. Results

**Figure 1:**
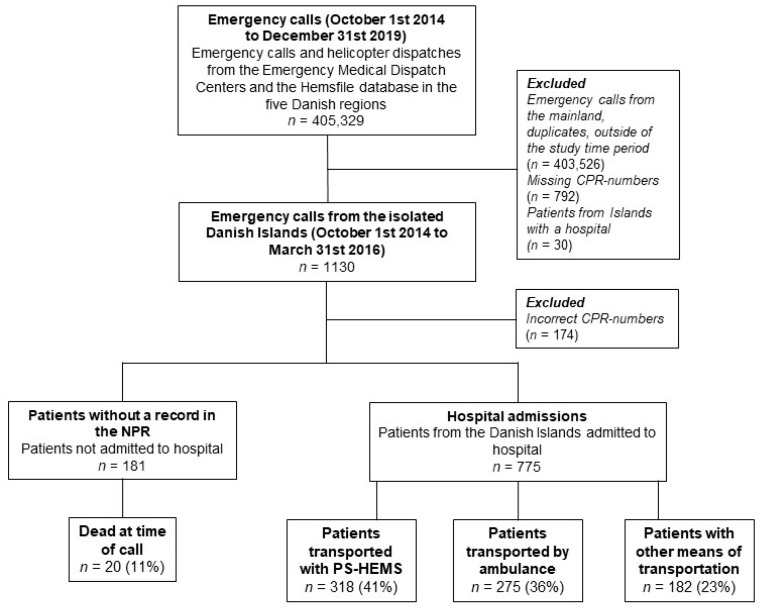
Flow chart of included patients with an emergency call from the isolated Danish Islands.

In the study period, we identified 1130 emergency calls from patients on the isolated Danish islands. A valid CPR-number was available in 956 cases (Figure 1).

Among these, 775 patients were admitted to the hospital with a registered admission course in the NPR. The HEMS transported a total of 318 patients; 275 patients were transported by a ground ambulance, and 182 patients were brought to the hospital by unknown means of transport. The median transport time from emergency medical system dispatch to arrival at the hospital was 83 min (IQR 66–104) for the patients flown with the HEMS and 90 min (IQR 45–144) for the patients transported by a ground ambulance (*p* = 0.26). The median transport time was 399 min (IQR 196–665) for the patients admitted to the hospital arriving by other unknown means of transportation (Table 1). 

The patients were predominantly males (58%), and the median age of the admitted patients was 65 years (IQR 49–75).

The overall 30-day mortality was 6.2% (CI: 4.6–8.1%). 

**Figure 2:**
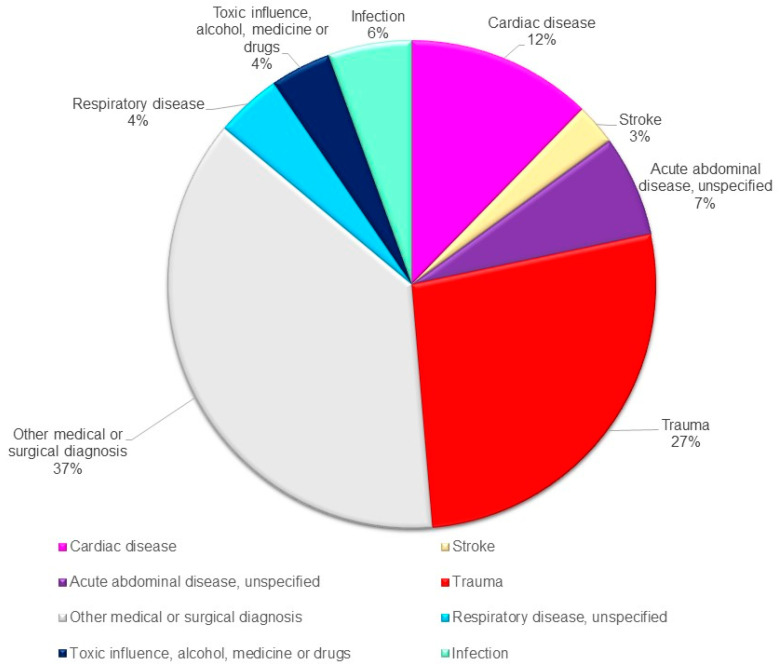
Diagnosis distribution of patients (n: 775) admitted to hospital from the Danish Islands.

The discharge diagnosis for the admitted patients from the isolated Danish islands was an unspecified medical or surgical diagnosis in 290 (37%, CI: 34.0–40.9%) of the cases registered, while trauma constituted 209 (27%, CI: 23.9–30.2%). In total, 34% (CI: 27.1–40.3%) of the trauma-related admissions were brought to the hospital by the HEMS and 41% (CI: 34.4–48.1%) by a ground ambulance. A total of 72 trauma patients (34 %, CI: 28.0–41.3%) were admitted to a hospital with a trauma center (Figure 2).

A total of 95 patients were diagnosed with acute cardiac disease. Fifty-seven patients (60%, CI: 49.4–70.0%) were transported by the HEMS, whereas 24 (25%, CI: 16.9–35.2%) patients were brought to the hospital by a ground ambulance. The proportion of acute cardiac disease among the HEMS patients was greater than among the patients brought to the hospital by a ground ambulance or other means of transportation (*p* = 0.001). Of the 95 patients with a diagnosis of acute cardiac disease, 58 (61%, CI: 50.5–70.9%) were transported to one of the five hospitals in Denmark with emergency PCI treatment (percutaneous coronary intervention) available, and 66% (CI: 51.9–77.5%) of these patients were transported by the HEMS (Table 2).

Out of the 21 patients with a diagnosis of stroke, 67% (CI: 43.0–85.4%) were brought to the hospital by the HEMS, and the proportion of stroke among the HEMS-transported patients was significantly higher than among the patients brought to the hospital by a ground ambulance or other means of transportation (*p* = 0.02). Of these 21 patients, 15 were brought to a hospital with thrombolysis treatment available. Sixty percent of the patients with a suspected stroke were brought by the HEMS to a hospital with thrombolysis treatment available. 

## 4. Discussion

We found 1130 emergency calls from the isolated Danish islands with 775 patient admissions recorded during an 18-month study period. Of these, 40% of the patients were transported by the HEMS and 36% were transported by a ground ambulance. The median admission time from the EMDC dispatch to arrival at the hospital was 83 min for the HEMS vs. 90 min by a ground ambulance, with no significant difference. The median admission time was 399 min by other means of transport. The patients with cardiac disease or stroke were predominately transported by the HEMS, whereas a larger proportion of the trauma patients were transported by a ground ambulance.

The main strength of this study was that we identified the patients included from the time of registration of their emergency contact. By using the data from the EMDCs, we were able to obtain the exact time of the emergency call and response dispatch. 

There are several limitations as it was not always possible to follow up with patients who had an emergency care contact during the study period. The triage and referral to the hospital is difficult through telephone visitation, and CPR numbers are not always registered correctly. It is, therefore, unclear what symptom presentation led to the dispatch of either the HEMS or ground ambulance. Some of the HEMS missions were not linked to an emergency contact. In total, 185 patients did not have a registered mode of transport, which makes it unclear if they were brought to the hospital by their own means or by ambulance/HEMS. These patients are still included in our data analysis in the unknown mode of transportation subgroup. 

In patients transported to one specific university hospital, we did not have the data of the mode of transport. Because this hospital is the biggest treatment center in its region, and because of its status as a university hospital, we assume that some of these patients were probably transported by the HEMS. Another limitation was the very few patients in some categories, which made it impossible to report on information about these patients because of data protection regulations. It was not possible to further differentiate between the degrees of trauma in the group of patients with injuries. The ICD-10 diagnosis coding system describes the anatomical location of injuries, as well as some information about the mechanism of injury and degree of bleeding. To assess the need for fast admission or expert prehospital care, we would have needed more information about the trauma severity, such as an injury severity score (ISS) [13]. 

The median time from emergency medical system dispatch to the patient arriving at the hospital was 83 min for the HEMS and 90 min for a ground ambulance, with no clinically important difference. A previous study on HEMS in Denmark has shown that HEMS in cases of emergency may be beneficial and was found to reduce time from dispatch to hospital admission by 34 min [14]. Contrarily, other studies of the Danish patient population with acute illness showed only a small or no time-benefit by using the HEMS for transport instead of a ground ambulance [15,16]. It thus seems debatable if the use of HEMS improves time to admission in emergencies in a country with only minor geographical obstacles, short distances to hospitals, and a well-developed infrastructure, but more research is needed within this field. The patient diagnoses recorded included more non-critical conditions than we expected. We identified 37% of patients with an unspecified diagnosis, which did not fit into the groups of more specific potentially time-critical illnesses or injuries. In terms of the mode of transport, including HEMS, ground ambulance, or unknown transportation, the proportion of patients without a specified critical illness were similar (33% vs. 30% vs. 26%). These proportions of unclear diagnoses are higher than in earlier studies [17]. 

In our study, the HEMS patients did have a high occurrence of critical illness, including cardiac disease and stroke. These conditions were more commonly recorded than expected in a general population of emergency patients on the mainland. A ground ambulance is the usual mode of transport for patients bound for PCI-treatment, and the HEMS is used for patients from the most distant parts of the country. This also explains the larger proportion of patients transported by HEMS with a stroke suspicion as these patients are also commonly transported to a hospital providing thrombolysis treatment. Ground ambulances are used when there is no obvious time benefit from using the HEMS, indicating that there might be an over-triage of patients from the Danish islands. Thus, a suspicion of a critical illness is enough to warrant a dispatch of the HEMS, allowing for a consultant anaesthesiologist on the HEMS to assess and treat the patient on the scene and during transport. 

We were not able to report on the number of cardiac arrests as the number of patients was too low to keep patient anonymity. There are around 5000 out of hospital cardiac arrests in Denmark yearly [18]. The number of citizens on the relevant islands comprise 0.4% of the Danish population, and, over a time period of 18 months, we would expect 24 cardiac arrests on these islands. We identified less than 10 cardiac arrests and found that 20 patients were dead at the time of the emergency call from an unknown cause of death.

In Denmark, there are 25,000 acute cardiac-related admissions yearly. We would, therefore, expect 150 patients with an acute cardiac diagnosis in our 18-month study period, where we identified 95 patients. Some patients with a suspected cardiac diagnosis could have been in the group of 181 patients who were not admitted to the hospital after the emergency call. 

In our study, we identified 61% of cardiac patients who were brought to a hospital with PCI treatment. For patients with cardiac symptoms, most patients would potentially benefit from being brought to a hospital with PCI treatment available. The time window for PCI treatment in patients with myocardial infarction is 120 min, and the patients of our study arrived after a median of 56 min, not including patient delay [16].

We found a low number of stroke patients in our study of 21 patients vs. the 72 that were expected during the study period. An explanation could be that some of these patients were not admitted to the hospital. The time window for thrombolytic treatment in patients with a suspected stroke is 4.5 h, and we found a median transport time to the hospital of 79 min, not including patient delay [15]. In Denmark, this indicates no apparent need for a mobile stroke unit (MSU) [19]. Conversely, in an international setting such as Australia, with largely differing logistical circumstances, an earlier study by Gardiner et al found the aeromedical retrieval of stroke patients to be outside of the transportation window for thrombolytic treatment [20].

Some patients who suffered from an acute cardiac or stroke diagnosis could have been registered with an unspecified diagnosis in this study.

Trauma patients in our study (*n* = 209) were mostly brought to the hospital by ground ambulance (41%). Only 72 out of the 209 trauma patients were admitted to a hospital with a trauma center. It is probable that most of the patients did not suffer from severe traumas but from minor injuries that could have been managed in a local hospital.

Because patients are triaged based on the suspicion of a condition and not an established diagnosis, it seems reasonable that a large proportion of the patients from islands were flown by HEMS (41%). Having an anaesthesiologist on scene is beneficial, but, for accurate measures of the efficacy of the HEMS, there is a need for more research in the overall triage of patients, which is an important area in the setting of prehospital care [21].

It is important to recognize the impact of the emergency care of the patients of isolated islands. Qualitative studies could be useful in describing the perception of the residents, and we would have liked to compare our results with the emergency care to isolated islands before the national HEMS was introduced. Further, we would have liked to know what other means of transportation these patients were brought to the hospital with as other means were the case for 23% of the patients in this study. 

## 5. Conclusions

In conclusion, we found that there was an approximately equal use of the HEMS and ground ambulance in the overall transport of patients from isolated Danish islands to the hospital, as well as no difference in the median transport time. The HEMS transported more patients with suspected stroke or cardiac disease. 

## Figures and Tables

**Table 1:** Characteristics of patients in need of emergency care from the isolated Danish Islands.

Numbers (*n*) with % or 95% CI, Medians with IQR	PS-HEMS	Ambulance	Other
Patient contacts, *n* (%)	318 (41%)	275 (36%)	182 (23%)
Males, *n* (%)	193 (61%)	171 (62%)	87 (48%)
Age, years, median (IQR)	66 (52–74)	66 (27–78)	61 (41–75)
30-day mortality, *n* (%, 95% CI)	22 (6.9%, 95% CI: 4.4–10.3%)	16 (5.8%, 95% CI: 3.4–9.3%)	10 (5.5%, 95% CI: 2.7–9.9%)
90-day mortality, *n* (%, 95% CI)	31 (9.8%, 95% CI: 6.7–13.5%)	23 (8.4%, 95% CI: 5.4–12.3%)	14 (7.7%, 95% CI: 4.3–12.6%)
Time interval from emergency medical system dispatch to arrival at initial hospital, median in minutes (IQR)	83 (66–104)	90 (45–144)	399 (196–665)
Diagnoses, *n*(%)			
Cardiac disease	57 (18%)	24 (9%)	14 (8%)
CNS related conditions	30 (9%)	33 (11%)	25 (14%)
Trauma	70 (22%)	86 (31%)	53 (29%)
Acute abdominal disease, unspecified	22 (7%)	22 (8%)	8 (4%)
Infection	19 (6%)	11 (4%)	13 (7%)
Respiratory disease	14 (4%)	8 (3%)	12 (7%)
Alcohol intoxication	1 (0%)	10 (4%)	8 (4%)
Other medical diagnoses	27 (8%)	18 (7%)	10 (5%)
Other, unspecified	78 (25%)	63 (23%)	39 (21%)

**Table 2:** Characteristics for patients admitted to hospital with acute cardiac disease, stroke, and trauma diagnoses.

	Acute Cardiac Disease		Stroke		Trauma	
Patients, *n* (%) HEMS (*N* = 318) Ambulance and other (*N* = 457)	95 57 (18%) 38 (8%)	*p =* 0.001	21 14 (4%) 7 (2%)	*p =* 0.02	209 70 (22%) 139 (30%)	*p =* 0.01
Age, years, median (IQR)	68 (57–75)		72 (61–77)		56 (25–71)	
HEMS: time to admission, minutes (IQR)	56 (30–94)	*p =* 0.6	78 (64–86)	*p =* 0.6	84 (75–109)	*p =* 0.4
Ground ambulance: time to admission, minutes (IQR)	50 (26–89)		79 (56–80)		82 (45–186)	

## Data Availability

The data that support the findings of this study are available from The Danish Health Data Authority in the following databases: The National Patient Registry and The Civil Patient Registration, but restrictions apply to the availability of these data, which were used under license for the current study and are not publicly available.
